# The Use of Corn Stover-Derived Nanocellulose as a Stabilizer of Oil-in-Water Emulsion

**DOI:** 10.3390/polym15030757

**Published:** 2023-02-02

**Authors:** Lingling Liu, Gina Gerard, Zimeng Peng, Zhile Yu

**Affiliations:** 1 Department of Agricultural and Biosystems Engineering, Iowa State University, Ames, IA 50010, USA; 2 Department of Food Science and Human Nutrition, Iowa State University, Ames, IA 50010, USA; 3 Department of Bioengineering, University of Pennsylvania, Philadelphia, PA 19104, USA

**Keywords:** TEMPO-CNF, essential oil, emulsion stabilizer, surfactant

## Abstract

Agricultural byproducts such as corn stover are widely available sources for preparation of nanocellulose, which is an emerging green chemical with versatile applications. In this study, corn stover-derived nanocellulose was prepared via bleaching, alkaline treatment, 2,2,6,6-tetramethylpiperidin-1-oxyl (TEMPO) oxidation, and ultrasonication. The as-prepared TEMPO-oxidized cellulose nanofibril (TEMPO-CNF) was characterized by transmission electron microscopy, UV-Vis spectrophotometry, rheometry, and zeta potential measurement. Droplet size, phase behavior, and thermodynamic stability of TEMPO-CNF stabilized oil-in-water emulsions were investigated. Results show that TEMPO-CNF with a width of 4 nm, length of 353 nm, and surface charge of 1.48 mmol/g COO^-^ can be prepared from corn stover. In addition, TEMPO-CNF can be used as an emulsion stabilizer for lemongrass essential oil loaded oil-in-water emulsion. This study is among the first to report that TEMPO-CNF improved the freeze-thaw stability of oil-in-water emulsions stabilized by small molecular weight surfactants (e.g., Tween 80).

## 1. Introduction

According to a US Department of Energy report, more than 75 million dry tons of corn stover are produced every year. Investigation of value-added use of corn stover can increase economic and environmental sustainability. With a cellulose content of ~45%, corn stover is a widely available cheap source for the preparation of nanocellulose. Nanocellulose is a cellulosic nanomaterial with at least one dimension less than 100 nm. Nanocellulose is biodegradable and biocompatible with negligible toxicity, and has been widely applied in fields such as pharmaceutics, packaging, textiles, and cosmetics [1,2,3,4,5,6]. Carboxylated nanocellulose (e.g., 2,2,6,6-tetramethylpiperidin-1-oxyl (TEMPO)-oxidized cellulose nanofibril (TEMPO-CNF)) is one of the most widely investigated forms of nanocellulose [7]. Currently, nanocellulose is often prepared from sources such as wood [8], cotton [9], and bacteria [10], while less research is focused on preparation of nanocellulose from agricultural byproducts such as corn stover/husk [11,12] and rice straw [13]. Corn stover-derived cellulose nanocrystals were reported to have a crystallinity of 55%, and a length and width of ~356 and ~7 nm, respectively [11]. Corn-husk-derived TEMPO-CNF had an average length and width of ~150 and ~20 nm, respectively [12]. Nonetheless, there are limited studies on the application of corn byproduct-derived nanocellulose [12]. In the proposed study, we investigated the physicochemical properties of corn stover-derived TEMPO-CNF and its application as an emulsion stabilizer.

Emulsions can be stabilized by low molecular weight surfactants (such as Tween 80) or high molecular weight biopolymers. For instance, small molecular weight synthetic surfactant Tweens have been widely used to stabilize emulsion products [14]. However, emulsions stabilized by Tween 80 were not stable against environmental stresses [15]. In addition, due to the demand for more natural alternatives, polysaccharides and proteins have also been widely studied as emulsion stabilizers [16]. In emulsions that are stabilized by finely divided solid particles (e.g., polysaccharides and proteins) (also called Pickering emulsions [17]), particles can adsorb at the oil–water interface and render good emulsion stability. Each emulsifier group has its advantages and shortcomings depending on its properties [16]. For instance, emulsions stabilized by polysaccharides often have a large particle size, although they are less affected by environmental stresses [18]. Therefore, it would be of interest to investigate the combination of binary emulsion stabilizers to render optimized properties on emulsion stabilization. 

Nanocellulose has been shown to be an effective Pickering emulsion stabilizer [19,20,21,22]. For instance, Mikulcová et al. [19] showed that oil-in-water emulsions stabilized by cellulose nanocrystals or microfibrillated cellulose was stable during storage and against mild centrifugation. In addition, larger than 95% encapsulation efficiency was achieved for emulsions loaded with antimicrobial oils up to 40 wt%. Foo et al. [21] also showed that Pickering emulsions stabilized by cellulose nanocrystals were stable for more than 6 months. TEMPO-CNF has also been studied as an emulsion stabilizer for dodecane in water emulsions and hexadecane in water emulsions [20,22]. However, so far knowledge is lacking regarding the performance of TEMPO-CNF as an emulsion stabilizer for essential oil-loaded emulsions. In addition, very few studies focused on the effect of a combination of nanocellulose and small molecular weight non-ionic surfactants on stabilizing emulsions. Therefore, the aim of this study was to investigate the phase behavior and droplet size of essential oil-loaded emulsions stabilized by TEMPO-CNF, as well as the thermodynamic stability of emulsions stabilized by both TEMPO-CNF and Tween 80. The effect of this binary emulsion stabilizer on emulsion stability is reported in this paper with the mechanisms explored. We report herein, for the first time, that TEMPO-CNF was capable of improving the freeze-thaw stability of Tween 80-stabilized oil-in-water emulsions. The findings will be useful for future application of TEMPO-CNF as stabilizers of essential oil-loaded emulsions and other emulsion products. This study also paves a new way for value-added use of corn byproducts. 

## 2. Materials and Methods

### 2.1. Materials

The corn stover sample was received from the Iowa State University BioCentury Research Farm (Boone, IA, USA) and ground into a size below 1/8 inch. All of the chemicals were purchased from VWR (Radnor, PA, USA) or Fisher Scientific (Pittsburgh, PA, USA).

### 2.2. Preparation of Nanocellulose from Corn Stover 

Corn stover was used as the source material for the preparation of nanocellulose according to the methods described by Costa et al. [11] with modifications. Specifically, corn stover was suspended in DI water and rinsed through a 75 µm sieve until the filtrate water was clear, followed by drying at 70 °C in an oven (QUINCY LAB, Burr Ridge, IL, USA), grinding in a grinder (YaeTek Swing Type Grain Mill Grinder, Shanghai, China) and passing through a 40-mesh sieve. A bleaching treatment (mixture containing 3 wt% sodium chlorite and 3 wt% acetic acid in DI water) was followed to remove lignin from corn stover with a solid–liquid ratio of 20:1 at 80 °C in a shaking water bath (Boekel Scientific, Feasterville-Trevose, PA, USA) for 2 h at 80 rpm. The delignified corn stover sample was filtered with a 25 µm sieve and washed until the affluent pH was close to the pH of DI water. The bleaching treatment was repeated, and the final delignified sample was dried at 60 °C in an oven, followed by grinding and sieving as described above. 

Alkaline treatment was followed by using 4 wt% NaOH with a solid–liquid ratio of 20:1 at 80 °C in a shaking water bath at 120 rpm for 2 h, followed by filtration with a 25 µm sieve and washing until the affluent was clear. Lastly, the corn stover sample was dried and ground with the above-mentioned grinder, followed by ball milling (Fritsch pulverisette, FRITSCH Milling and Sizing, Inc., Pittsboro, NC, USA) at 100% speed for 10 min to further reduce the particle size. The sample was then passed through a 40-mesh sieve and used for TEMPO oxidation treatment.

After bleaching and alkaline treatment, the corn stover sample was used to prepare TEMPO-CNF using the TEMPO/NaBr/NaClO oxidation system, according to Okita et al. [23] with modifications. Briefly, TEMPO and NaBr were used as catalysts, with NaClO as the primary oxidant. Specifically, 3 g corn stover sample was suspended in 300 mL DI water containing 0.048 g TEMPO and 0.30 g sodium bromide. Then, NaClO (10 mmol/g cellulose) was dropwise added to the suspension under magnetic stirring and the addition was completed within 10 min. The pH of the suspension was maintained at 10.0~10.1 with the addition of 0.5 M NaOH, until no consumption of NaOH was observed. Then, 0.3 g NaBH_4_ was added to the suspension with continuous stirring. After 3 h, the suspension was centrifuged (Eppendorf 5430R, Enfield, CT, USA) at 7745 *g* for 10 min at 4 °C. The pellet was washed with DI water, followed by centrifugation for 3 times, then TEMPO-CNF gel was obtained. The solid content of the concentrated gel was determined by measuring the moisture content of the sample. The diluted gel sample was prepared by diluting the obtained gels with DI water, followed by ultrasonication using a 500 W ultrasonicator (Fisher Scientific, Hampton, NH, USA) for 2 h in an ice bath, centrifugation at 7745 *g* for 10 min at 4 °C and collection of the supernatant. Centrifugation was applied to remove any impurities released from the ultrasonic probe, or to remove any un-fibrillated and partly fibrillated fractions if present.

The color of the corn stover sample after each treatment was recorded by a colorimeter (3nh, Shenzhen THREENH Technology Co., Ltd., Shenzhen, China). The yields of the corn stover sample after each treatment were calculated by the following equations. Specifically, step yields and overall yields were both calculated.

(1) $$\%\;Step\;yield_{i}=\frac{Dried\;sample\;weight\;after\;a\;certain\;treatment_{i}}{Dried\;sample\;weight\;before\;a\;certain\;treatment_{i}}\times 100\%$$

(2) $$\%\;Overall\;yield_{After\;treatment\;n}=Step\;yield_{1}\left(\%\right)\times Step\;yield_{2}\left(\%\right)\ldots .\times Step\;yield_{n}\left(\%\right)$$

where *n* = 1, 2, 3, 4.

### 2.3. Conductometric Titration of TEMPO-CNF

The carboxyl content of the prepared nanocellulose was determined according to the methods described by Liu et al. [24] with modifications. Specifically, 50 mL of the diluted gel sample (0.1 % (*w/v*)) was added to a beaker; then, the pH of the suspension was adjusted to 2.0 by addition of 0.1 M HCl to make the carboxylate groups in protonated forms. The suspension was allowed to stand for 15 min; then, 1.0 mL of 1.0 mM NaCl was added, and the suspension was stirred for 90 min before titration. Titration was performed with the addition of 0.1 M NaOH at the rate of 0.1 mL per 30 s. The conductivity value of the suspension during each addition of NaOH was recorded using an Oakton PC 700 pH meter (Cole-Parmer, Vernon Hills, IL, USA), and the titration was continued until the pH of the suspension reached 10.5. The carboxylate content of the sample was calculated from the conductivity and pH curves. Specifically, the titration curves show the presence of a strong acid and a weak acid, which correspond to the excess of HCl and the carboxylate content, respectively. The carboxyl content was calculated using the equation below.

(3) $$N=\frac{\left(V_{1}-V_{0}\right)\times C_{N_{a}OH}}{m}\;\;\;$$

where *N* represents the carboxyl content (mmol/g), *V*_1_ and *V*_0_ represent the equivalent volumes (mL) of added NaOH solution, *C_NaOH_* represents the concentration (mol/L) of NaOH solution, and *m* represents the dried mass (g) of sample.

### 2.4. Characterization of TEMPO-CNF

The dimensions of the as-prepared TEMPO-CNF were determined using transmission electron microscopy (TEM) according to the methodology described in Costa et al. [11]. Specifically, the diluted gel sample was observed using a 200 kV JEM-2100 scanning/transmission electron microscope (STEM) (JEOL Ltd., Akishima, Tokyo). The optical transmittance of the prepared TEMPO-CNF gel (0.1 wt%) was scanned from 200 to 1100 nm using a UV-Vis spectrophotometer (Azzota Scientific, Claymont, DE, USA). The zeta potential of diluted TEMPO-CNF sample was determined using a Zetasizer Nano ZS instrument (Malvern Instruments Ltd., Worcestershire, UK). The rheological behavior of TEMPO-CNF was determined using the method in our previous publication [25]. In brief, the suspension of TEMPO-CNF at different concentrations was measured using a Discovery HR-2 rheometer (TA instruments, Newcastle, DE, USA) equipped with a DIN Concentric Cylinder with bob and cup geometry (bob diameter = 28 mm, cup diameter = 30.4 mm). Samples were conditioned at 37 °C for 5 min after loading, then a flow ramp test over a shear rate range of 1–100 s^−1^ and a 180 s period (sampling interval of 1 s/pt) was performed at 37 °C. Three tests were performed for each sample and the viscosity readings versus shear rate were recorded. 

### 2.5. Nanocellulose-Stabilized Pickering Emulsion

#### 2.5.1. Preparation of Nanocellulose-Stabilized Pickering Emulsion

To prepare nanocellulose-stabilized Pickering emulsions, TEMPO-CNF suspensions at varying concentrations were added to lemongrass essential oil (EO) with magnetic stirring, followed by ultrasonication with a 500 W ultrasonicator for 5 min. The EO-loaded emulsion contained 2.5 wt% EO and certain concentrations of TEMPO-CNF (0, 0.1, 0.3, 0.5, and 0.7 wt%). The emulsion samples were stored at room temperature (away from light) and observed for phase separation over time. 

#### 2.5.2. Determination of Emulsion Droplet Size 

Images of the TEMPO-CNF-stabilized emulsions were captured with an optical microscope (50W Halogen Trinocular Microscope, AmScope, United Scope LLC., Irvine, CA, USA) immediately after sample preparation. Specifically, 10 µL of undiluted sample (right after sample preparation) was pipetted onto a microscope slide and covered with a coverslip. Images were taken under 40× objective magnification. Droplet size of the emulsion was determined from the images using Image J 1.53t software (Wayne Rasband and contributors, National Institutes of Health, Washington, DC, USA). 

### 2.6. Oil-in-Water Emulsion Stabilized by TEMPO-CNF and Tween 80

#### 2.6.1. Preparation of Oil-in-Water Emulsions Stabilized by TEMPO-CNF and Tween 80

Emulsion samples with the presence of both TEMPO-CNF and a low molecular weight surfactant (i.e., Tween 80) were also prepared. The EO-loaded emulsions contained certain concentrations of lemongrass EO (1, 2.5, and 5 wt%), certain concentrations of Tween 80 (EO/Tween 80 ratio 1:3), and with or without the presence of TEMPO-CNF (0.3 wt%). Emulsions were prepared by slowly adding Tween 80 to EO with stirring, then the aqueous phase (containing nanocellulose and water) was added with stirring, followed by ultrasonication with a 500 W ultrasonicator for 5 min.

#### 2.6.2. Thermodynamic Stability Testing of Oil-in-Water Emulsion

Several thermodynamic stability tests, such as centrifugation and freeze-thaw tests of emulsion samples, were performed to assess their stability. For the centrifugation test, emulsion samples were centrifuged at 10,000 rpm (Eppendorf 5418, Enfield, CT, USA) for 15 min at 25 °C and observed for phase separation (if any). During the freeze-thaw test, emulsion samples were stored for 2 cycles with each cycle of storage at −20 and 25 °C for 48 h each, then observed for phase separation (if any). 

## 3. Results

### 3.1. Yield and Color of Corn Stover Sample after Each Treatment

The appearance of the corn stover sample after each treatment is shown in Figure S1 (Supplementary Materials). Table 1 shows the color parameter of the corn stover sample after each treatment. Specifically, the washed and ground corn stover sample shows a yellow color (corresponding to the smallest L* value but largest a* and b* values). After the removal of lignin during bleaching treatment, the sample appeared white (corresponding to the increased L* value but decreased a* and b* values). With the removal of hemicellulose during the following alkaline treatment, the sample appeared more whitish. Finally, after TEMPO oxidation, the freeze-dried TEMPO-CNF appeared totally white (corresponding to the largest L* value but smallest a* and b* values).

Table 1 shows that the overall yields decreased after each treatment. The chemical composition of corn stover was reported to be ~45% cellulose, ~28% hemicellulose, and 6.8%–19.6% lignin [11,26]. The washing step had a yield of ~87.4%. The loss during the washing process is probably due to the removal of dirt and other water-soluble extractives, as well as small particles that have a size below 75 µm. The largest sample loss occurred after bleaching treatment. The loss of corn stover samples during bleaching treatment is mainly due to the removal of lignin, polyphenol, and protein [26], as well as small particles with a size below 25 µm. The bleaching treatment incorporated in this study resulted in a whitish sample, indicating that lignin was mostly removed. Sample loss during alkaline treatment is probably due to the removal of hemicellulose and ash [27], as well as small particles with a size below 25 µm. During the TEMPO oxidation treatment, water-insoluble fractions were recovered with a yield of ~87%. This is similar to that reported in literature, where the recovery rate of samples was generally higher than 80% after TEMPO oxidation [28].

### 3.2. Physicochemical Properties of TEMPO-CNF

**Figure 1:**
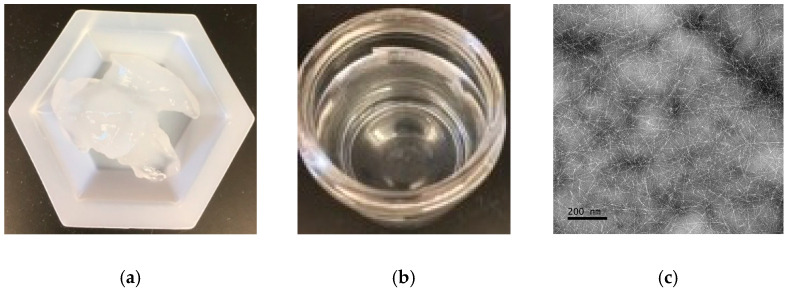
Macroscopic and microscopic images of TEMPO-CNF prepared from corn stover. (**a**) The as-prepared TEMPO-CNF concentrated gel; (**b**) 10-times diluted gel suspension after sonication; (**c**) TEM image of TEMPO-CNF.

Figure 1a shows that TEMPO-CNF prepared from corn stover presents as a gel. The as-prepared concentrated TEMPO-CNF gel looks like a paste, but it has a low solid content of 3.80% ± 0.08%. Figure 1b shows that diluted gel suspension after sonication is transparent. Specifically, according to Figure S2 (Supplementary Materials), light transmittance of 0.1 wt% TEMPO-CNF is almost 100% across the wavelength of 400–1100 nm. This is similar to that reported by Hu et al. [29]. At the same gel concentration (0.1 wt%), the light transmittance at 600 nm of TEMPO-CNF derived from corn stover (this study) is similar to that prepared from softwood bleached kraft pulp or microcrystalline cellulose (both ~99% light transmittance) [30], but slightly higher than that extracted from jute bast fibers (~90%) [31]. The similarities in the light transmittance between our study and that reported in Zhou et al. [30] may be due to the same TEMPO oxidation process involved, while the differences in the light transmittance between our study and Cao et al. [31] may be due to the different parameters of the TEMPO oxidation process.

Figure 1c shows that TEMPO-CNF derived from corn stover has a needle-like shape. TEMPO-CNF had a width of ~4.0 nm, length of ~353 nm, and length/width ratio of ~88. This is very similar to that reported in other studies. For instance, TEMPO-oxidized nanocellulose prepared from softwood bleached kraft pulp had a width of 3.5 to 3.6 nm and average lengths of ∼200 nm [30]. TEMPO-CNF prepared from wood had a homogeneous width of ~3 nm and high aspect ratios (>150) [32]. 

According to Table 2, corn-stover derived TEMPO-CNF has a zeta potential of -65 mV, indicating that it is colloidally stable. The highly negative surface charge of TEMPO-CNF achieved during the oxidation process is also reported in other studies [23]. For instance, TEMPO-CNF derived from multiple cellulose sources including softwood and hardwood had a zeta potential of ~−75 mV [23]. TEMPO-CNF prepared from softwood bleached kraft pulp or microcrystalline cellulose had a zeta potential of −54 and −60 mV, respectively [30]. 

In addition, it is determined from Figure S3 (Supplementary Materials) that corn-stover derived TEMPO-CNF has a surface charge of 1.48 mmol -COONa/g dry sample. The anionic surface charge of TEMPO-CNF fibrils due to carboxyl groups results in electric repulsion, and therefore has a high absolute zeta potential value. This is similar to that published in literature. Specifically, the carboxylate content of TEMPO-CNF derived from corn stover (this study) is lower than that of softwood (1.65 mmol/g) and hardwood (1.69 mmol/g) bleached kraft pulps, but higher than that of cellulose sources from cotton (1.36 mmol/g), *Acetobacter xylinum* bacteria (1.05 mmol/g), Tunicates of *Halocynthia roretzi* (0.59 mmol/g), and whole plants of *Cladophora* sp. (0.52 mmol/g) [23]. With 10 mmol/g NaClO in the TEMPO/NaBr/NaClO in water at pH 10, the carboxylate group content varies (0.52 –1.7 mmol/g) according to the cellulose origin [23]. At carboxylate contents higher than 1 mmol/g, highly transparent and viscous gels can be obtained after applying gentle mechanical disintegration of the fibrils [33].

**Figure 2:**
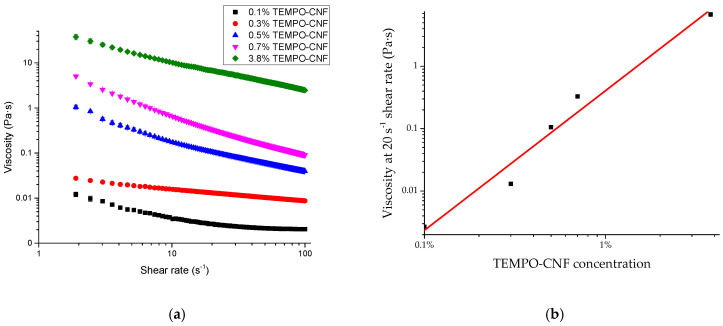
Viscosity of corn-stover derived TEMPO-CNF. (**a**) Viscosity as a function of shear rates; (**b**) viscosity as a function of TEMPO-CNF concentration at selected shear rate (20 s^−1^).

The viscosity of TEMPO-CNF at different concentrations is shown in Figure 2a. It shows a shear thinning behavior, which was also observed in our previous study [25,34]. Viscosity at a 20 s^−1^ shear rate (η) as a function of TEMPO-CNF concentration (C) is shown in Figure 2b and was fitted by the power law model (log_10_ η = a + blog_10_ C), which is similar to that of our previous studies [25,34]. The fitting parameters are as follows: a = 4.09, b = 2.24, and adjusted R^2^ = 0.96. At a shear rate of 20 s^−1^, viscosity increases at higher TEMPO-CNF concentrations. Specifically, the viscosity of TEMPO-CNF at 3.81 wt% is 6.75 Pa·s, which is much higher than that of 0.7 wt% (0.33 Pa·s), 0.5 wt% (0.105 Pa·s), 0.3 wt% (0.013 Pa·s), and 0.1 wt% (0.003 Pa·s). Based on the fitting parameters, the viscosity of corn-stover derived TEMPO-CNF (our study) is slightly lower than that of wood-derived TEMPO-CNF (produced by USDA’s Forest Products Laboratory), as wood-derived TEMPO-CNF had fitting parameters of a = 5.45 and b = 2.42, as shown in our previous study [34]. As the carboxyl contents for corn stover-derived TEMPO-CNF (our study) and wood-derived TEMPO-CNF (USDA’s Forest Products Laboratory) were both ~1.5 mmol/g, it is postulated that the differences in viscosity may be due to the material sources and/or preparation procedures. However, the viscosity of corn stover-derived TEMPO-CNF (our study) was higher than another kind of wood-derived TEMPO-CNF (with different oxidation and separation procedures as compared to TEMPO-CNF from USDA’s Forest Products Laboratory) [35], reflecting that the preparation procedures influenced the product’s viscosity. The relationship between viscosity and polymer concentration in this study was similar to that reported for hydroxyethyl cellulose, for which power law models were fitted, and the fitting parameters varied according to the polymer molecular weight and the polymer concentration regime [36].

### 3.3. TEMPO-CNF Stabilized Pickering Emulsion

Lemongrass essential oil loaded emulsions with or without the presence of TEMPO-CNF did not show any phase separation right after sample preparation (result not shown here). However, after 24 h of storage at room temperature (absence from light), the emulsions prepared without TEMPO-CNF or with low concentrations of TEMPO-CNF (below 0.5 wt%) underwent phase separation. No phase separation was observed in emulsions stabilized by TEMPO-CNF at concentrations above 0.5 wt%. The percentages of the creaming layers in the emulsions are shown in Table 3. The overall trend is that the creaming layer percentage decreased with the increase in TEMPO-CNF concentration. Similar observations have been reported by others using TEMPO-CNF as Pickering emulsion stabilizers [20,22]. Specifically, Goi et al. [20] showed that TEMPO-CNF could stabilize emulsions against creaming by adsorbing onto oil droplets and forming a network structure in the water phase at concentrations above 0.15 wt%; but when the emulsion was diluted with water, it underwent creaming and phase separation after 24 h. In addition, Gestranius et al. [22] showed that TEMPO-CNF stabilized Pickering emulsions containing 1% TEMPO-CNF and 20% oil underwent creaming and phase separation within 24 h after emulsification. 

A similar phenomenon of concentration-dependent emulsion stability against creaming was also observed for Pickering emulsions stabilized by other biopolymers such as starch nanocrystals [37], chitin nanocrystals [38], microcrystalline cellulose [39], nanoparticles from insoluble soybean polysaccharide [40], and heat-induced soy protein nanoparticles [41]. It was shown that, at low particle concentrations, an oil-in-water Pickering emulsion stabilized by nanoparticles derived from insoluble soybean polysaccharides also showed layer separation, while at high particle concentrations, no phase separation was observed [40].

**Figure 3:**
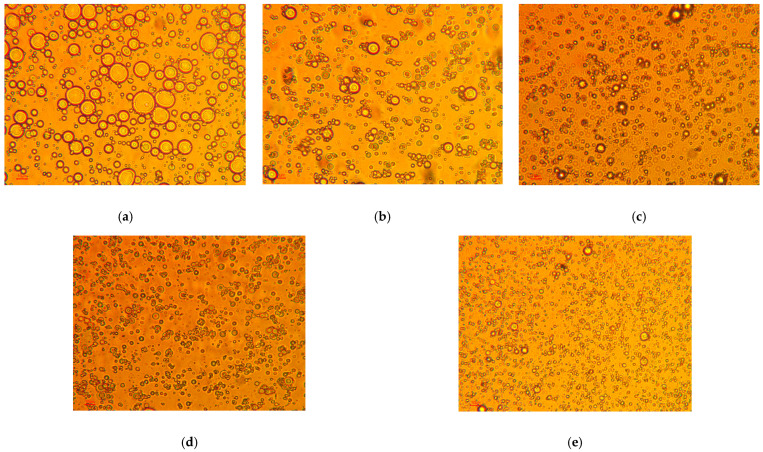
Optical microscopic images of lemongrass essential oil loaded emulsions stabilized by TEMPO-CNF at varying concentrations of (**a**) 0%, (**b**) 0.1 wt%, (**c**) 0.3 wt%, (**d**) 0.5 wt%, and (**e**) 0.7 wt% TEMPO-CNF. Note: All the scale bars are 10 µm.

**Figure 4:**
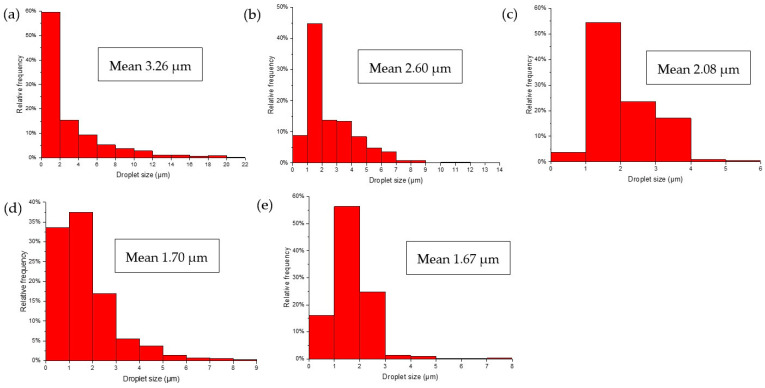
Droplet size distribution of lemongrass essential oil loaded emulsions stabilized by TEMPO-CNF at varying concentrations of (**a**) 0%, (**b**) 0.1 wt%, (**c**) 0.3 wt%, (**d**) 0.5 wt%, and (**e**) 0.7 wt% TEMPO-CNF (corresponding to microscopy images in Figure 3).

Figure 3 shows the optical microscopy images of the emulsions stabilized at varied concentrations of TEMPO-CNF. As shown in Figure 3, all the emulsions flocculated right after preparation. This finding is similar to that reported by Gestranius et al. [22], who showed that TEMPO-CNF could stabilize emulsions against coalescence but not flocculation. Both Figure 3 and Figure 4 show that, in the emulsion systems using TEMPO-CNF as stabilizer, the overall trend is that the average droplet size of emulsion decreased with the increase in TEMPO-CNF concentration. Specifically, the average droplet size decreased from 3.26 to 1.67 µm when increased TEMPO-CNF concentration from 0 wt% to 0.7 wt%. Solid particle concentrations are shown to influence the droplet size and viscosity of emulsion systems [42], which is common for Pickering emulsion systems. The emulsification performance of particles can be reflected by its Sauter mean diameter (d_32_), and, in general, smaller d_32_ corresponds to better emulsification performance [40]. It was shown that the surface mean diameter (d_32_) of hexadecane-in-water emulsions decreased as the bacterial cellulose nanocrystal concentration increased until it reached a minimum value [43]. The effect of solid particle concentrations on emulsion droplet size has also been reported by Chen et al. [44] and Yang et al. [40]. For instance, with an increase in particle concentration (from 0.1 to 1.2 wt%), a progressive decrease in emulsion droplet size was observed in Pickering emulsions stabilized by unmodified or octenylsuccinic anhydride-modified cellulose nanocrystals [44].

It was reported that solid particles can irreversibly adsorb on droplet surface. At low particle concentrations, emulsion droplets are only partially covered by the particles, thus, the system is not stable. However, at higher particle concentrations, excess particles form a network structure surrounding droplets and enhance viscosity, thus improving the system stability [42]. As shown in Section 3.2, higher TEMPO-CNF concentrations corresponded to higher viscosity. Therefore, at higher TEMPO-CNF concentrations, more TEMPO-CNF nanofibrils can surround oil droplets and form a network structure.

It was also reported that TEMPO-CNF can form nanosized emulsion droplets. Specifically, emulsion droplets with a size as low as 350 nm were achieved by cotton TEMPO-CNF [45]. TEMPO-CNF forms an entangled system of nanometer-size emulsion droplets due to the electrostatic repulsion of carboxyl groups [45]. Nanocellulose with varying aspect ratios has distinct mechanisms of stabilizing lipid emulsions [25]. Specifically, cellulose nanofibrils with large aspect ratios formed an interconnected network around oil droplets, while cellulose nanocrystals with small aspect ratios wrapped outside each individual droplet surface, and TEMPO-CNF behaved between cellulose nanofibrils and cellulose nanocrystals. Schemes of oil-in-water emulsions containing nanocellulose were reported in our previous study [25]. 

### 3.4. Emulsions Stabilized by TEMPO-CNF and Tween 80

Emulsions stabilized by TEMPO-CNF (0.3 wt%) and a low molecular weight surfactant (i.e., Tween 80) were also studied. Thermodynamic stability tests were performed, and comparisons were made among emulsions stabilized by Tween 80 with or without the presence of TEMPO-CNF. Results are shown in Table 4. 

Table 4 shows that lemongrass essential oil loaded emulsion stabilized by Tween 80 was stable against the centrifugation test with or without the presence of TEMPO-CNF. Without the presence of TEMPO-CNF, all the formulated emulsions stabilized by Tween 80 only were not stable against two cycles of freeze-thaw tests. With the addition of 0.3% TEMPO-CNF, all the formulated oil-in-water emulsions were stable against two cycles of freeze-thaw tests. Combining the results of the centrifugation and freeze-thaw stability tests, the stable formulations include 0.3% TEMPO-CNF, and 1–5% lemongrass essential oil.

The inability of Tween 80 to stabilize the oil-in-water emulsions against the freeze-thaw process was also reported by Ariyaprakai and Tananuwong [15]. Specifically, Tween 80 stabilized corn oil-in-water emulsions coalesced and destabilized during the freeze-thaw process. In general, small molecular weight surfactants such as Tween 80 provide a relatively thin interfacial layer around droplets, and thus cannot provide enough stability for the emulsions against the freeze-thaw process [15]. Emulsion droplets covered by thick interfacial membranes can better protect crystal penetration and partial coalescence [46]. For instance, Pickering emulsions stabilized by starch granules can form a dense layer around droplets, provide resistance against crystal penetration, and thus increase the stability of emulsions during the freezing process [46]. In addition, proteins can provide a thicker interfacial membrane and thus better stability for the emulsions against the freeze-thaw process [15]. Based on the findings in Section 3.3, it is postulated that TEMPO-CNF behaves similarly to protein by providing thick interfacial membranes surrounding emulsion droplets, thus enhancing the freeze-thaw stability of oil-in-water emulsions.

Polymers with ice recrystallization inhibition activity have been shown to enhance the freeze-thaw stability of materials [47]. TEMPO-CNF has been reported to exhibit ice recrystallization inhibition activity [48]. Specifically, ice recrystallization in 0.01 M NaCl was effectively inhibited by 2.0 mg/mL TEMPO-CNF [48]. It is postulated that the ice recrystallization inhibition activity possessed by TEMPO-CNF may be one of the mechanisms involved in its ability to improve the freeze-thaw stability of emulsions. Specifically, the ability of TEMPO-CNF to reduce the amount of frozen water, modify the ice crystal structure, and decrease the collision frequency of droplets renders it ability to improve the freeze-thaw stability of oil-in-water emulsions in this study. 

To the best of our knowledge, the current work is the first to report the ability of TEMPO-CNF to improve the freeze-thaw stability of Tween 80-stabilized oil-in-water emulsion. Based on the findings of this study, it was postulated that both TEMPO-CNF and Tween 80 adsorbed on the oil-water interface. Synergistic effects may occur between TEMPO-CNF and Tween 80 for stabilization of oil-in-water emulsions. Similarly, synergistic effects have been reported between nanocellulose and saponin (a natural emulsifier) [49]. In addition, in binary emulsion systems stabilized by pectin and Tween 80, it was reported that both pectin and Tween 80 adsorbed on the oil-water interface [50]. 

According to Dickinson [51], the presence of polysaccharides in emulsions stabilized by small molecular emulsifiers may result in better or worse stability against creaming, depending on the type and concentration of the polysaccharide. For instance, in Tween 20 stabilized oil-in-water emulsions, the addition of an increased xanthan concentration resulted in a complex pattern of the serum volume fraction, with the percentage of the serum phase first increasing, reaching a maximum value, and then decreasing to 0 [51]. It was postulated in this study that 0.3 wt% TEMPO-CNF may enhance the thermodynamic stability of Tween 80 stabilized oil-in-water emulsions.

## 4. Conclusions

In this work, corn stover-derived TEMPO-CNF gel with a solid content of ~3.8 wt% was prepared, characterized, and studied as an emulsion stabilizer. The as-prepared TEMPO-CNF had a width of 4 nm, length of 353 nm, and surface charge of 1.48 mmol/g COO^-^. At 0.1 wt%, the colloidal suspension of TEMPO-CNF had a light transmittance of ~100% at a wavelength of 400–1100 nm, indicating that the suspension is transparent. At higher concentrations, the colloidal suspension of the prepared TEMPO-CNF had higher viscosity. Lemongrass essential oil loaded emulsions stabilized by TEMPO-CNF at concentrations above 0.5 wt% did not undergo any phase separation after 24 h of storage at room temperature. Decreased emulsion droplet size was observed in emulsions stabilized by higher TEMPO-CNF concentrations. Specifically, an average droplet size of 1.67 µm was observed in emulsions stabilized by 0.7 wt% TEMPO-CNF. The presence of TEMPO-CNF did not affect the stability of Tween 80 stabilized oil-in-water emulsions against centrifugation, that is, both types of emulsions did not form phase separation after centrifugation. Furthermore, to the best of our knowledge, this is the first study to report that TEMPO-CNF possesses the capability to improve the freeze-thaw stability of Tween 80 stabilized oil-in-water emulsions. Findings from this study lay a foundation for further application of nanocellulose as an emulsion stabilizer against environmental stresses. The developed emulsion systems are also consistent with growing interest in using natural substances to make stable emulsion products.

## Figures and Tables

**Table 1:** Yield and color of corn stover sample after each step of treatment.

	Washing	Bleaching Treatment	Alkaline Treatment	Oxidation Treatment
Step yield (%)	87.4 ± 9.0	55.1 ± 1.7	66.3 ± 4.8	86.9 ± 11.2
Overall yield (%)	87.4 ± 9.0	48.2 ± 5.2	31.9 ± 4.1	27.7 ± 5.1
L*	24.3 ± 3.2	34.0 ± 1.0	34.6 ± 4.8	35.4 ± 1.5
a*	4.4 ± 0.3	3.1 ± 0.1	3.3 ± 0.7	2.1 ± 0.1
b*	12.3 ± 0.06	9.8 ± 0.2	7.0 ± 0.3	4.4 ± 0.4

**Table 2:** Physicochemical properties of TEMPO-CNF.

Physicochemical Properties	Value
Length (nm)	353 ± 116
Width (nm)	4.0 ± 0.6
Length/width ratio	88 ± 32
Zeta potential (mV)	−65 ± 3
Concentrated gel solid content	3.80% ± 0.08%
Light transmittance at 600 nm (0.1 wt% gel suspension)	100.0% ± 0.2%
Carboxyl group (mmol/g)	1.48 ± 0.09

Note: The length and width information of TEMPO-CNF derived from corn stover were calculated based on multiple TEM images collected.

**Table 3:** Creaming layer percentage of emulsions stabilized by TEMPO-CNF after 24 h of storage at room temperature.

TEMPO-CNF Concentration (%)	Creaming Layer Percentage ^1^ (%)
0	N/A ^2^
0.1	10.9 ± 0.2
0.3	9.4 ± 1.4
0.5	0
0.7	0

Note: ^1^ Creaming layer percentage is calculated based on the ratio of the height of the creaming layer and the total height of the sample. ^2^ The sample separated into an oil layer and a clear serum layer.

**Table 4:** Phase behavior of emulsions stabilized by Tween 80 (Oil/Tween 80 ratio 1:3) with or without the presence of TEMPO-CNF against centrifugation and freeze-thaw cycle tests.

Lemongrass Essential Oil Concentration (*w/w*)	TEMPO-CNF Concentration (*w/w*)	After Centrifugation	After 2 Freeze-Thaw Cycles
1%	0	No phase separation	Phase separation
2.5%	0	No phase separation	Phase separation
5%	0	No phase separation	Phase separation
1%	0.3%	No phase separation	No phase separation
2.5%	0.3%	No phase separation	No phase separation
5%	0.3%	No phase separation	No phase separation

## Data Availability

The data presented in this study are available on request from the corresponding author.

## Supplementary Materials

The following supporting information can be downloaded at: https://www.mdpi.com/article/10.3390/polym15030757/s1, Figure S1: Macroscopic images of corn stover samples after each treatment. (a) Washed and grinded corn stover sample; (b) corn stover sample after bleaching treatment; (c) corn stover sample after bleaching and alkaline treatment; (d) freeze-dried TEMPO-CNF derived from corn stover. Figure S2: UV-Vis transmittance spectra of TEMPO-CNF at 0.1 wt%. Figure S3. Determination of the carboxyl content of TEMPO-CNF by using the conductometric titration method.
