# Supplementary material for: The Use of Corn Stover-Derived Nanocellulose as a Stabilizer of Oil-in-Water Emulsion
Source: Polymers (Basel). 2023 Feb 2;15(3):757. doi: 10.3390/polym15030757 (PMC9920403; doi:10.3390/polym15030757)
Supplement: Supplementary file 1 [file polymers-15-00757-s001.zip › polymers-2106781-supplementary.pdf]

Supplementary Material

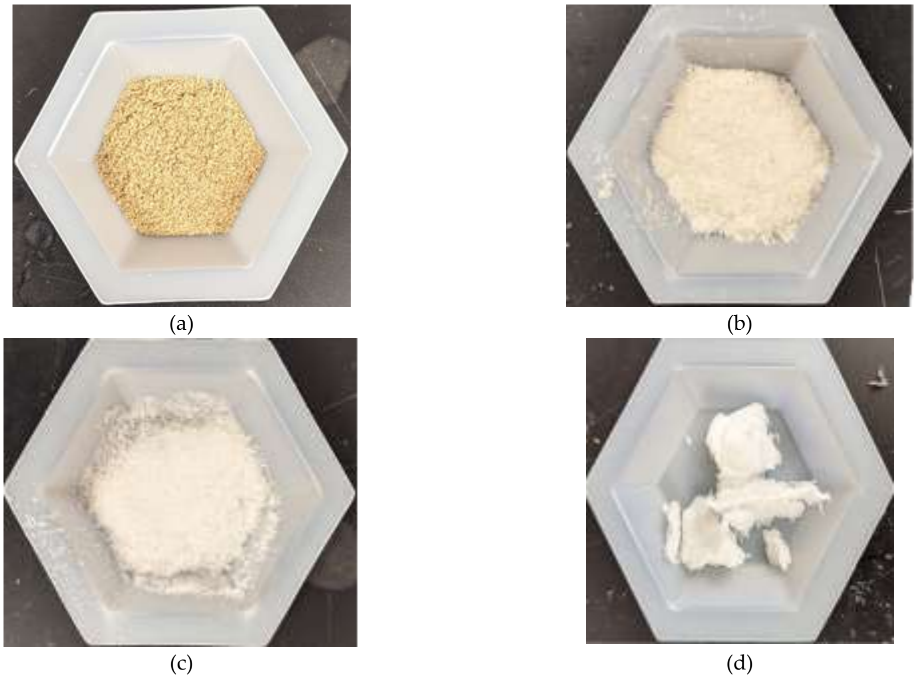

Figure S1. Macroscopic images of corn stover samples after each treatment. (**a**) Washed and grinded corn stover sample; (**b**) corn stover sample after bleaching treatment; (**c**) corn stover sample after bleaching and alkaline treatment; (**d**) freeze-dried TEMPO-CNF derived from corn stover.

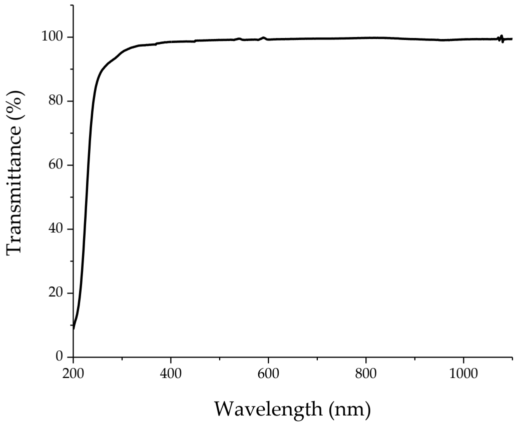

Figure S2. UV-Vis transmittance spectra of TEMPO-CNF at 0.1 wt%.

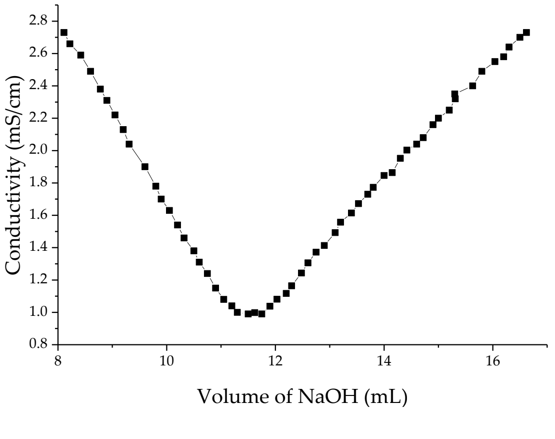

Figure S3. Determination of the carboxyl content of TEMPO-CNF by using the conductometric titration method.
